# Neutralization of Dengue Virus Serotypes by Sera from Dengue-Infected Individuals Is Preferentially Directed to Heterologous Serotypes and Not against the Autologous Serotype Present in Acute Infection

**DOI:** 10.3390/v13101957

**Published:** 2021-09-29

**Authors:** Heidi Auerswald, Simone Kann, Leonard Klepsch, Janne Hülsemann, Ines Rudnik, Sebastian Schreiber, Philippe Buchy, Michael Schreiber

**Affiliations:** 1 Department of Virology, Bernhard Nocht Institute for Tropical Medicine, 20359 Hamburg, Germany; hauerswald@pasteur-kh.org (H.A.); simone.kann@medmissio.de (S.K.); leonardklepsch@gmail.com (L.K.); janne.huelsemann@posteo.de (J.H.); ines.rudnik@haw-hamburg.de (I.R.); sebastian@schryber.com (S.S.); 2 Virology Unit, Institut Pasteur in Cambodia, Phnom Penh 12201, Cambodia; philippe.x.buchy@gsk.com; 3 GlaxoSmithKline, Vaccines R&D, Singapore 139234, Singapore

**Keywords:** dengue virus, neutralization, serotype, antigen assay, domain 3, E protein

## Abstract

Sequential infections of humans by the four different dengue serotypes (DENV-1–4) lead to neutralizing antibodies with group, cross, and type specificity. Virus neutralization of serotypes showed monotypic but mostly multitypic neutralization profiles due to multiple virus exposures. We have studied neutralization to heterologous, reference DENV serotypes using paired sera collected between days 6 and 37 after onset of fever. The DENV-primed neutralization profile of the first serum sample, which was monitored by a foci reduction neutralization test (FRNT), was boosted but the neutralization profile stayed unchanged in the second serum sample. In 45 of 47 paired serum samples, the predominant neutralization was directed against DENV serotypes distinct from the infecting serotype. Homologous neutralization studies using sera and viruses from the same area, 33 secondary sera from DENV-1 infected Cambodian patients and eight virus isolates from Cambodia, showed that the FRNT assay accurately predicted the lack of a predominant antibody response against the infecting DENV-1 serotype in contrast to FRNT results using the WHO set of DENV viruses. This report provides evidence that DENV-primed multitypic neutralizing antibody profiles were mainly boosted and stayed unchanged after secondary infection and that DENV neutralization was predominantly directed to heterologous DENV but not against the infecting homologous serotype.

## 1. Introduction

Dengue viruses (DENV), as part of the family *Flaviviridae*, represent an antigenic subgroup in the genus Flavivirus [1]. They are characterized by a distinctive genetic variability and are classified into four subspecies, designated DENV-1 to DENV-4. Subtyping of DENV has been historically performed by serotyping—a method in which DENVs are antigenically differenced based on reactions with DENV type-specific antibodies by plaque reduction neutralization tests (PRNT) [2], indirect immunofluorescence (IFA) [3,4], or enzyme-linked immunosorbent assays (ELISA) [5]. Today, serotyping has been developed based on viral RNA detection [6]. Thus, instead of antigenic differences, genetic differences are used to differentiate between the four so-called DENV serotypes. Nevertheless, our understanding on dengue virus variation might be inchoate, since DENVs cluster more based on their antigenic properties [7,8] rather than as distinct genetically defined serotypes [9]. In general, the effect of antigenic variation by amino acid substitutions cannot easily be predicted from virus sequences. Single substitutions can improve antibody binding, but sometimes, multiple amino acid substitutions are necessary to produce the same viral phenotype and vice versa. Therefore, differences in viral phenotypes and the capacity to neutralize DENV must be tested by antibody neutralization assays using monoclonal, polyclonal, or serum antibodies [10,11,12].

In addition to the classification of DENV, it is an important task to study the potential of neutralizing antibodies in sera from DENV-infected patients against the different DENV subtypes, the so-called neutralization profile to DENVs. It is a matter of common knowledge that infection with a distinct DENV leads to a humoral immune response that protects patients against the infection with the homologous type but not against infection by heterologous virus [13,14,15,16]. Such a matching, type-specific homotypic response can be clearly seen in the primary infection of formerly naïve patients [17,18]. The situation becomes tremendously complex when the type-specificity of neutralizing antibodies is studied in secondary DENV infection in comparison to the infecting virus [19]. After secondary infection, individuals develop homotypic as well as multitypic neutralizing antibodies to DENVs in which the multitypic response, or multitypic neutralization profile, is directed against virus subtypes they had probably never been exposed to [12]. Thus, the anti-DENV humoral response is a combination of group-, cross-, and type-specific antibodies all binding to a variety of DENVs [20]. Detailed studies of polyclonal responses in human sera showed that the E protein is a target for group-, cross-, and type-specific antibodies whereby the latter, type-specific antibodies, represent only a very small minority of the total anti-E antibody response [20,21].

It is suggested that after secondary infection, the type-specific antibody response will become predominant, shifting to type-specificity directed against the infecting virus. However, it is thought that second serum samples, regularly collected between days 6 and 8 after the onset of fever, are collected too early to see those type-specific antibodies arising. The maturation of immune response and the difference in terms of reactivity between strains due to antigenic differences was previously studied in experimental infections. Monkeys were challenged with a variety of different virus isolates, and their immune response was measured. Monkey antisera could generally neutralize the autologous virus much better than heterologous types [22]. Interestingly, after sequential infection by different DENV serotypes, no solid immunity to dengue could be achieved [23]. The latter is an interesting study, since it is an autologous challenge and protection experiment that mimics more precisely natural infection in humans. It indicates that the DENV-primed immune response caused by the first infection is not sufficiently supplemented by the second virus to establish full protection.

A model of B cell maturation following sequential DENV infections proposed by Patel et al. [12] suggests that after secondary infection, the cross-specific response will be significantly boosted. Thus, with each new DENV infection, the ratio of type-specific and cross-reactive antibodies is getting smaller, leading to a decrease in type-specific responses and an increase in the cross-specific antibody population [12]. From these experiments, it can be concluded that the analysis of the DENV-specific neutralization profile in sequential serum samples will show a general boost of neutralization titers. However, this anti-DENV boost will not be accompanied by a shift to the highest neutralization titers directed against the last current DENV serotype.

This report aims to provide data on the DENV-specific neutralization profiles from heterologous and homologous neutralization experiments using serum samples collected between 3 and 37 days after onset of dengue fever. In addition, two indirect diagnostic assays (ED3 ELISA and ED3 dot assay) were performed to detect serotype-specific antibody responses in sera from DENV-infected patients, which were examined for a unique serotype by a direct virus assay (RT-PCR). From the comparison of data received from direct and indirect serotyping studies, it seems that the neutralizing antibody profile remains unchanged with the highest antibody response or neutralizing activity directed against a DENV serotype not in consistency with the serotype detected in acute infection.

## 2. Materials and Methods

### 2.1. Cell Culture

VeroB4 cells were used for DENV cultivation as well as for the 90% foci reduction neutralization test (FRNT^90^). VeroB4 cells were cultured in Dulbecco’s modified Eagle’s medium (DMEM), supplemented with 10% FCS (fetal calf serum), 2 mM glutamine (PanBiotech, Aidenbach, Germany), 1% antibiotic solution (5000 U/mL penicillin, 5.000 µg/mL streptomycin; Gibco, USA) at 37 °C and 5% CO_2_.

### 2.2. Viruses

The DENV strains used for neutralization tests are summarized in Table 1. All viruses were cultivated in VeroB4 cells. Virus culture supernatants were harvested by centrifugation and concentrated with 8% PEG 8000 overnight at 4 °C. Viruses were precipitated by centrifugation at 1500× *g* for 30 min. The virus-containing pellets were suspended in DMEM and stored at −80 °C until use.

### 2.3. Serum Specimens

Dengue positive serum samples (*n* = 504) were provided by the Institut Pasteur in Cambodia (IPC). Sera were formerly characterized by RT-PCR, IgM MAC-ELISA, and HIA [17]. The use of stored and partially anonymized samples for research purposes was approved by the Cambodian National Ethics Committee. Paired serum samples (*n* = 7) from Colombia were collected from patients who were tested positive for dengue by a commercial RT-PCR (RealStar Dengue RT-PCR Kit 1.0, altona Diagnostics, Hamburg, Germany) in a study at the Hospital Rosario Pumarejo de Lopez in Valledupar, Colombia, which was approved by the local ethic commission. 

### 2.4. Foci Reduction Neutralization Test

The foci reduction neutralization test (FRNT^90^) was carried out as described earlier [24]. In brief, flat-bottom 96-well microplates were seeded with VeroB4 cells (4 × 10^4^ per well) 24 h before infection. Serum dilutions starting at 1:10 were prepared in DMEM and added to equal volumes of virus supernatants. Virus–serum mixtures were incubated for one hour at 37 °C and finally added to VeroB4 cells. After virus infection, a semi-solid overlay (0.8% methyl cellulose, DMEM, 10% FCS) was added, and the microplates were incubated for three days at 37 °C followed by a treatment with formaldehyde solution (3.7% in PBS (137 mM NaCl, 2.7 mM KCl, 10 mM Na_2_HPO_4_, 1.8 mM KH_2_PO_4_, pH 7.4)). Microplates were washed with PBS followed by a PBS/0.5% Triton X-100 treatment for 20 min at room temperature and washed again with PBS/10% FCS. Infected cells were detected using anti-DENV mouse hyperimmune ascites fluids (IPC, Phnom Penh, Cambodia). Staining was performed using anti-mouse IgG antibody conjugated to horseradish peroxidase (Bio-Rad, München, Germany) and 3, 3′, 5, 5′-tetramethylbenzidine as substrate (SureBlue™ TMB 1-component microwell peroxidase substrate, medac, Wedel, Germany). The stained cells (foci) were counted immediately, and the endpoint titers were expressed as reciprocal of the highest serum dilution showing ≥ 90% reduction in foci counts (FRNT^90^ titer) compared to wells without serum. All sera were tested in triplicate. The DENV serotype-specific antibody response was considered as the highest FRNT^90^ titer to one of the DENV serotypes.

### 2.5. Maltose Binding Protein–ED3 Fusion Proteins

The ED3 domains were expressed in *E. coli* BL21 as a fusion to maltose-binding protein (MBP). The ED3 and ED3s domains of the WHO (World Health Organization) recommended DENV strains were cloned as described earlier [24]. The COL (Colombian dengue strains) ED3 domains were cloned using the assembly PCR method. The list of synthetic oligonucleotides (TIB Molbiol, Berlin, Germany) for the COL MBP-ED3 antigens is shown in Table A1. A mixture of 2 µL for each oligonucleotide (10 µM), 0.6 mL dNTP (10 mM), 6 µL GC-buffer, and 0.3 µL *Taq*-DNA-Polymerase (New England Biolabs, Frankfurt, Germany) in a total volume of 30 µL was assembled using 10 temperature cycles of 98 °C (90 s), 56 °C (120 s), and 72 °C (120 s). From the assembly mixture, 0.4 µL were given to 20 µL GC-buffer, 1 µL *Taq*-DNA-Polymerase (New England Biolabs, Frankfurt, Germany), 5 µL forward (BamHI) primer (10 µM), 5 µL reverse (HindIII) primer (10 µM), and 2 µL dNTP (10 mM) in a total volume of 100 µL. The BamHI/HindIII DNA fragment was amplified using 45 temperature cycles of 98 °C (50 s), 56 °C (60 s), and 72 °C (120 s). Amplified DNA was extracted from a 0.8% agarose gel (QIAquick Gel Extraction Kit, QIAGEN, Hilden, Germany). The purified DNA was cloned into the pCR2.1 plasmid (TA-Cloning Kit, Invitrogen, Dreieich, Germany) according to the manufacturer’s recommendations. Positive clones were selected by white/blue screening on ampicillin/X-gal/IPTG plates and controlled by sequence analysis (LGC Genomics, Berlin, Germany). After BamHI and HindIII cleavage, the ED3 fragments were cloned into the MBP expression plasmid (pMal-p4x), and expression in *E. coli* BL21 was carried out as described earlier [24]. In brief, a volume of 2.7 L of dyt-medium supplemented with 300 µg/mL ampicillin, 0.5 mM IPTG, and 0.2% glucose was inoculated 1:100 with an overnight culture and incubated at 37 °C. Bacteria were harvested and treated with lysozyme (5 mg/mL) prior to sonication. Clear lysates were obtained after centrifugation at 15,000× *g* (Eppendorf 5810R, rotor FA-45-6-30, Hamburg, Germany). The MBP–ED3 fusion proteins were purified from clear lysates using amylose resin affinity chromatography according to the manufacturer’s recommendations (New England Biolabs, Frankfurt, Germany).

### 2.6. ED3 Dot Assay

A very detailed description of the ED3 dot assay was done earlier by Auerswald et al. [24]. Purified MBP-ED3 or MBP-ED3s fusion proteins (1 mg/mL) were used for the ED3 dot assay and the modified, SDS denatured form of ED3, designated mED3 or mED3s, was produced by adding SDS to a final concentration of 1% to the respective ED3 constructs, heated to 95 °C for 10 min and dotted, together with the other ED3 antigens (0.5 µL, 1 mg/mL), onto nitrocellulose strips (2.7 mm × 115 mm, BA85, Schleicher & Schuell, Dassel, Germany). After the dotting procedure, the test strips were transferred to a 30-well incubation tray (Viramed, Planegg, Germany) and were incubated with 1 mL PBST blocking buffer (PBS, 0.05% Tween 20, 5% low fat milk) for 1 h at room temperature. The blocking buffer was replaced against the serum solution (1:100 in PBST, 5% low fat milk) and incubated for 2 h on a shaker followed by three wash cycles with PBST. The detection of bound human antibodies was carried out using an anti-human IgG antibody conjugated to horseradish peroxidase (Bio Rad, Hercules, CA, USA) diluted 1:1000 in PBST blocking buffer. After 1 h of incubation, the test strips were washed again three times with PBST followed by three wash cycles with PBS. Bound antibodies were visible as dots after 10–20 min incubation with 4-chloro-1-naphtol (4CN) solution, a freshly prepared mixture of 200 mL PBS, 100 µL H_2_O_2_ (30% stock solution, Merck, Darmstadt, Germany), and 40 mL 4CN solution (0.3% in methanol). The intensity of the dots was analyzed by an in-house made purpose-built dot analyzing software (BlotLog) [24] or by using the universal graph digitizer software UN-SCAN-IT (Silk Scientific, Orem, UT, USA).

### 2.7. ED3 ELISA

The MBP–ED3 antigen concentration was adjusted to 2 µg/mL with bicarbonate/carbonate buffer (100 mM, pH 9.6). Plates with the diluted antigens, 100 µL per well (96-well plate, Maxisorb, Greiner Bio-One, Frickenhausen, Germany), were sealed and incubated at 8 °C overnight. Sera, diluted 1:100, were pre-incubated to MBP (75 µg/mL) at 8 °C overnight. Prior to antibody testing, each antigen-coated well was additionally filled with 300 µL blocking buffer (PBS, 5% low fat milk), and the plates were incubated for 1 h at room temperature. Plates were washed three times with PBST, and in each well, 100 µL of the serum–MBP mixture was added and incubated at room temperature for 1 h. After antibody binding, the plates were again washed three times with PBST, and 100 µL of HRP-conjugated goat anti-human anti-IgG antibody (Bio-Rad, Feldkirchen, Germany) diluted 1:2000 were added to each well and incubated for 1 h. Plates were washed three times with PBST, followed by three wash cycles with PBS. To each well, 50 µL of TMB substrate (KPL SureBlue, medac, Wedel, Germany) was added. The color reaction was stopped after 20 min by adding 50 µL of TMB BlueSTOP solution (KPL, medac, Wedel, Germany). The intensity of the color reaction was measured at 640 nm.

## 3. Results

### 3.1. Comparison of Direct DENV Detection and Serotyping Using the ED3 Dot Assay

Second serum samples were selected from the IPC Biobank with defined DENV serotype verification by RT-PCR. For each of the four DENV serotypes, 97−100 sera were chosen to be analyzed for the presence of DENV type-specific antibodies by the ED3 dot assay. The assay is using two antigens, recombinant mED3 and mED3s fused to maltose-binding protein (MBP).

**Figure 1:**
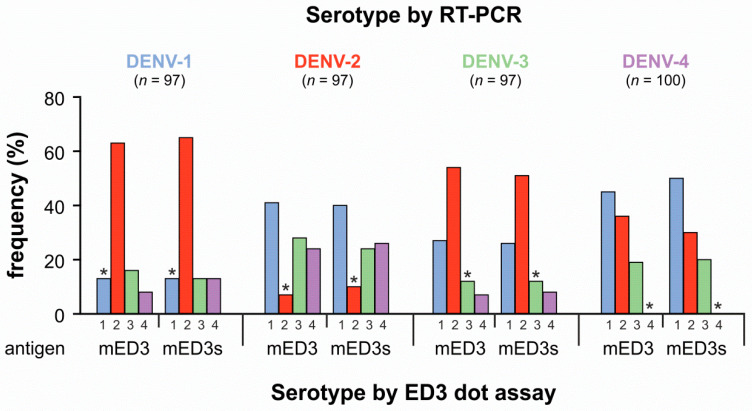
ED3-specific antibody detection by ED3 dot assay in RT-PCR defined sera. Sera were collected from Cambodian patients infected by DENV-1 (*n* = 97, blue), DENV-2 (*n* = 97, red), DENV-3 (*n* = 97, green), or DENV-4 (*n* = 100, purple). The ED3 dot assay was performed using mED3 and mED3s antigens representing ED3 amino acid sequences of DENV reference strains as given in Table 1. X-axis, numbers indicate DENV serotype tested by mED3 or mED3s antigens. *, indicating matching results.

From the DENV-1 infected group, all 97 sera showed antibody responses to one of the four serotypes with the most frequent response to the antigens representing the DENV-2 strain 16681 (Figure 1). More than 60% of the DENV-1 sera showed type-specific antibody response against the mED3 and mED3s DENV-2 antigens. Thus, for the majority of the DENV-1 sera, the predominant antibody response was directed against a DENV serotype different to the serotype present during infection. That the predominant antibody response was directed against a different serotype and not against the infecting serotype was also shown in the three other groups that were RT-PCR positive for DENV-2, DENV-3, and DENV-4.

In the DENV-2 group, the predominant antibody response was against the DENV-1 serotype, which was followed by DENV-3 and DENV-4. Only 7−10% of the sera showed a matching DENV-2 response. In the DENV-3 group, the matching antibody response was in third place with a frequency of 12%, followed by DENV-4 serotype specific neutralization with a frequency of 7−8%. Thus, in total, 80% of the sera showed a non-matching antibody response with antibody responses highest for DENV-1 and DENV-2. In the DENV-4 group, predominant neutralization was observed mainly against DENV-1 followed by DENV-2 and DENV-3. None of the sera showed a matching antibody response in the DENV-4 group; thus, a 100% mismatch between the infecting serotype and serotype-specific antibody was observed. Altogether, in all four groups, more than 85% of the sera showed a different serotype compared to the serotype identified during DENV infection (DENV-1 > 85%; DENV-2 > 90%, DENV-3 > 88%, DENV-4 = 100%).

### 3.2. Neutralization of DENV by Sera from RT-PCR Diagnosed DENV Cases

By using 40 DENV-positive paired serum samples from Cambodian patients, ten pairs for each DENV serotype as confirmed by RT-PCR, we wanted to investigate what is the highest neutralization titer (FRNT^90^) to one of the reference serotypes (listed in Table 1). In these experiments, the time frame between the onset of fever and the collection of the second serum was 7 ± 1.4 days.

When sera were tested for virus neutralization, the highest FRNT^90^ titers were observed against DENV serotypes that were not identical to the serotype present in acute infection (Table 2, marked in gray). For example, the paired sera (no. 01, Table 2) were from a DENV-1 infected patient, but the first serum showed the highest titer for neutralization against DENV-2 (1:5120) in contrast to DENV-1, where the titer was significantly four 2-fold dilutions lower (1:320). In the first serum, the serotype-specificity of the antibodies to DENV in decreasing titer magnitude was DENV-2 (1:5120), DENV-4 (1:2560), DENV-3 (1:640), and with the weakest FRNT^90^ titer for DENV-1 (1:320). In addition, in the second serum, the highest neutralization titer was observed for DENV-2 (1:20,480) followed by DENV-4 (1:5120). Thus, neutralization was highest for DENV-2 in both sera, and the neutralization profile to DENV-1-4 was unchanged, but neutralization titers were boosted. Such non-matching results between serotype by RT-PCR and serotype by FRNT^90^ were seen in 38 out of the 40 paired sera. Only two paired samples, no.09 and no.15 (Table 2), showed a matching antibody response to the corresponding reference strains.

The serotype-specificity of the neutralizing antibody response to DENV serotypes in the 80 Cambodian serum samples is summarized in Table 3 together with FRNT^90^ results from 39 Colombian RT-PCR positive sera. The data indicate that in 112 out of the 119 sera, the serotype-specific antibody response was against a heterologous DENV serotype, not in accordance with the DENV serotype identified in acute infection.

### 3.3. Neutralization of Heterologous and Homologous Serotypes Using DENV-1 Positive Sera

From 33 DENV-1 positive Cambodian patients, sufficient volumes of the second serum sample were available from the IPC Biobank to perform neutralization assays using the twelve DENV serotypes as listed in Table 1. The aim was to compare the neutralization of DENVs between the reference panel of viruses (REF) and the two sets of viruses isolated from patients in Cambodia, which were designated IPC A and IPC B (Table 1). The FRNT^90^ titers for each serum sample against the twelve DENV serotypes are given in Table 4. Clear matching FRNT^90^ titers to the infecting DENV-1 were seen with the reference DENV-1 strain 16007 in only six of the 33 serum samples. All other sera showed predominant neutralization to heterologous viruses or were double-positive. The neutralizing-specificity observed by using the three sets of viruses is summarized in Table 5. We observed a clear shift to non-DENV-1 specificity with the IPC A and IPC B set of viruses. In addition, the DENV-1 neutralizing specificity observed in double-positive sera (DENV-1 + 2; DENV-1 + 4, Table 4) completely disappeared after testing antibody-dependent neutralization with IPC A and IPC B viruses. Thus, using homologous viruses for virus neutralization (viruses and sera collected at the same time and in the same endemic region), it becomes more persuasive that serum neutralization is not predominantly directed against the infecting, the last current serotype that was detected in the serum sample. 

### 3.4. Neutralizing Antibody Responses and Serotype Specificity in Late Serum Samples

**Figure 2:**
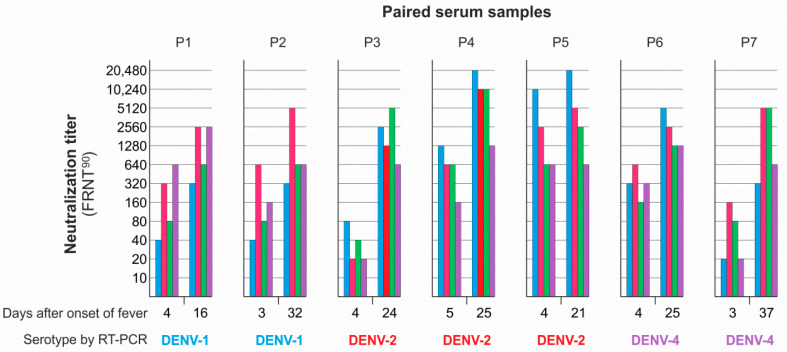
Neutralization of DENV-1–4 reference strains by paired serum samples. Sera were collected from Colombian patients infected by DENV-1, DENV-2, and DENV-4 as diagnosed by RT-PCR. Viruses (REF set) used for foci reduction neutralization test (FRNT^90^): blue, DENV-1 (16007); red, DENV-2 (16881); green, DENV-3 (H87); purple, DENV-4 (H241).

In this experiment, we tested paired sera collected at a longer time interval, as shown in Figure 2. Second sera were collected between days 16 and 37 after onset of fever. All first sera were from early days of infection (days 3−5), and in these sera, DENV was detected and classified by a commercial RT-PCR diagnostic kit (RealStar Dengue RT-PCR Kit 1.0, altona Diagnostics, Hamburg, Germany). All sera were tested for antibody-dependent neutralization using the reference panel of DENV serotypes (Table 1), and the FRNT^90^ was carried out in the same way as for the Cambodian paired sera, as shown in Table 2.

In agreement with the previous FRNT^90^ data, the predominant neutralizing activity in the second serum sample from the Colombian patients was not directed against the serotype that was detected in acute infection. Thus, even 16 to 37 days after onset of fever, a matching, predominant neutralizing antibody response to the infecting DENV serotype could not be detected. As shown before (Table 2), we also observed a significant boost of neutralization titers but the neutralization profile was uniform in six (P1−P5, P7) of the seven paired serum samples. In the P6 samples, predominant neutralization shifted from DENV-2 to DENV-1, but neutralization specificity for the infecting DENV-4 was significantly lower in both the first and second serum samples (P6, Figure 2).

**Figure 3:**
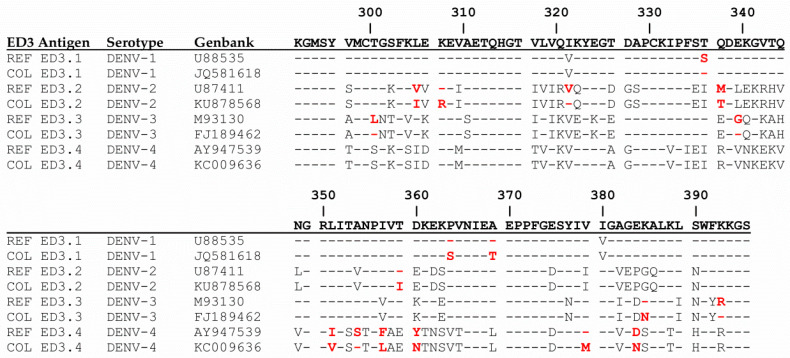
ED3 amino acid sequences used for ED3 ELISA and ED3 dot assay. Sequence differences between the same serotype are marked in red. Amino acid differences between REF:COL for DENV-1, S339T, P364S, A369T; for DENV-2, V308I, I310R, V324I, M340T, T359I; for DENV-3, L303T, G342E, K385N, R393K; for DENV-4, I351V, F357L, Y360N, V379M, D384N.

To test antibody responses against homologous epitopes, we developed two sets of antigens for the ED3 dot assay and ED3 ELISA. One set designated REF ED3.1−4 represented the ED3 domains of the reference panel of viruses and another set, designated COL, represented the ED3 domains of Colombian DENV strains. Colombian ED3 sequences were taken from the NCBI database, and those showing the highest degree of diversity to the REF ED3 sequences were chosen for cloning and expression. The ED3 sequences and the amino acid differences between REF ED3and COL ED3 antigens are depicted in Figure 3.

**Figure 4:**
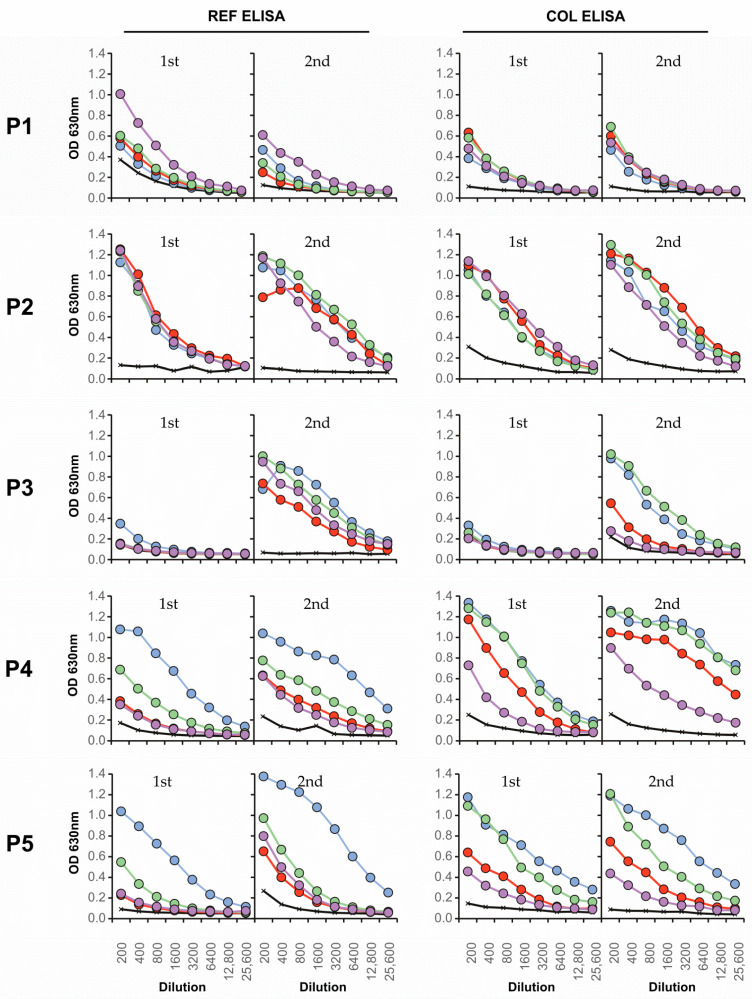

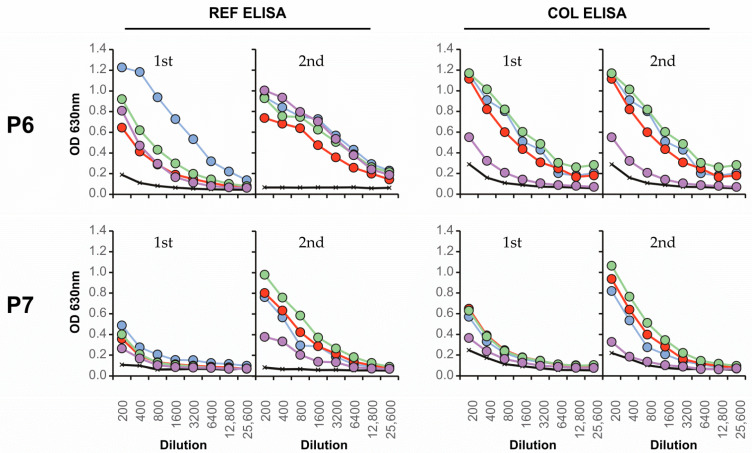
ED3-specific antibody detection by ED3 ELISA. Sera were collected from Colombian patients infected by DENV (P1–P7, Figure 1). Blue, ED3.1 (DENV-1); red, ED3.2 (DENV-2); green, ED3.3 (DENV-3); purple, ED3.4 (DENV-4). REF, antigens with ED3 sequences of a panel of reference strains. COL, antigens with ED3 sequences of Colombian strains. ED3 sequences and amino acid differences between REF and COL antigens are shown in Figure 2.

The ELISA results showed that using the REF ED3 and COL ED3 antigens, serotype-specific reactivities were detectable (Figure 4). For serum samples P1, RT-PCR confirmed that for DENV-1, a serotype-specific but weak response against REF ED3.4 was detected in the first sample. No serotype specificity nor antibody boost was observed with the COL ED3.1–4 antigens. For P2, RT-PCR confirmed for DENV-1, we detected an overall weak boost to all eight ED3 antigens but with no serotype specificity for DENV-1. For P3, which was RT-PCR positive for DENV-2, we observed an overall boost of antibody responses against the REF ED3.1–4 antigens and could detect a clear serotype-specific reaction to COL ED3.1 and COL ED3.3. For P4 and P6, which were RT-PCR positive for DENV-2 and DENV-4 respectively, we detected REF ED3.1 and COL ED3.1, -.2, and -.3 specific responses. For P5, which was RT-PCR positive for DENV-2, the sera showed a DENV-1 specific response. For P7, which was RT-PCR positive for DENV-4, both set of antigens showed responses to ED3.1, -.2, and -.3 but not against ED3.4.

P1−P7 sera were also tested by the ED3 dot assay for the detection of serotype-specific antibody responses (serum dilutions 1:100). Results of the ED3 dot assay were compared to the results obtained using ED3 ELISA, FRNT^90^, and RT-PCR (Table 6). The best match between the antibody detection assays was observed for the FRNT^90^ and the ED3 Dot Assay. Especially the data from the COL ED3 Dot Assay were very close to the FRNT^90^ results. ED3 Dot Assay antigens used for antibody detection are presented on nitrocellulose strips in a denatured form in contrast to native ED3 antigens used in the ED3 ELISA. Thus, using denatured as well as native antigens gave no RT-PCR matching results. Interestingly, COL ED3 antigens when tested by ELISA showed a much better match to FRNT^90^ data compared to the REF ELISA data. This indicates that for ELISA assays, it seems to be important to use antigens that represent the actual antigen variation of DENVs present in the endemic region. For the ED3 Dot Assay, using denatured antigens, this seems to be less important. Taken together, with all three assays, no predominant antibody response directed against the infecting DENV serotype was detectable in late sera collected between 16 and 37 days after onset of fever.

## 4. Discussion

The development of antibodies is crucial to prevent DENV entry into permissive cells through a process called antibody-dependent neutralization. Humans who become infected with a virus are starting to produce antibodies precisely binding to a variety of epitopes on multiple virus proteins, but only a subset of these antibody species have virus-neutralizing capacity. Neutralizing antibodies prevent reinfection, and this immunity lasts sometimes for the whole life. This situation becomes more complex when a virus has the capacity to easily escape from neutralization by antigenic drift or shift.

In the case of the DENV, cluster-, group-, cross-, and type-specific antibodies are developed, which can have non-neutralizing activity or can even enhance viral infection through antibody-dependent enhancement (ADE). The neutralizing antibody species plays an important role to protect humans from natural DENV infection. Therefore, measuring neutralizing antibody responses evolved by natural infection is an important task for the understanding of how the virus is priming and boosting its host. In a first attempt, we have studied antibody responses in 391 well-defined sera by a fast and simple screening test for neutralizing antibodies [24]. By using antigens representing domain III of the protein E from a reference panel of DENVs (REF), we have observed that neutralizing antibodies were mainly directed against a DENV serotype different from the infecting one. To further support this observation, we have performed virus neutralization tests with again four panels of sera that were characterized for infecting serotypes by RT-PCR. To compare results between laboratories, the panel of reference DENV strains was used for foci reduction neutralization assay (FRNT). In agreement with these recommendations, we also observed that the predominant antibody response against the infecting serotype was not a perfect match when we analyzed sera collected shortly after the onset of fever (1st sera) as well as sera collected about 7 days after onset of fever (2nd sera). Using the DENV reference panel, in one of 40 of sera, we found a predominant neutralization to the infecting virus. All other sera showed predominant neutralization to one of the DENVs that was not the infecting one. By analyzing the 2nd serum samples, we observed an overall boost of the DENV-neutralizing activity but no specific boosting directed against the infecting DENV serotype.

The data so far support the theory of antigenic sin, but ED3 antigens or viruses used for neutralization might be different in sequence to viruses that had originally stimulated the patients’ immune response. To analyze neutralization more specifically, we have used DENV serotypes isolated from Cambodian patients changing the design of the neutralization assay from a heterologous virus–serum assay to a more homologous assay. From the IPC Biobank, serum samples are typically available in small amounts, which are still suitable to perform diagnostic tests such as the ED3 dot assay that only needs ten microliter of serum. For the standard neutralization assay, the situation is different, since much more serum is needed (≥ 500 µL). We could create a panel of 33 sera from DENV-1-confirmed cases to perform the homologous neutralization tests. We used eight DENV strains, two for each of the four DENV serotypes, isolated from Cambodian patients and the four reference strains for comparison. As shown before, we observed no match between the infecting DENV type and the predominant titer of neutralization. Especially with the Cambodian strains, we observed a complete mismatch, indicating that the theory that the neutralization of DENV is directed to a serotype involved in a previous infection and not to the autologous one is even more obvious when the homologous virus is tested. When we included in the assay a homologous strain (i.e., a virus that was isolated during the same epidemic season that the sera were collected), then the ability of neutralization tests to identify DENV-1 serotype was significantly lower than with the reference strains.

After subsequent DENV infection, what is measured in the early, first serum sample, unless it was collected a bit late, around day 4 to 5, would reflect past infection. It is actually common sense that in the second sera, a polyclonal heterotypic response with the highest titer directed against the most recent past infecting serotype or even the first one will be detectable. This follows the idea of original antigenic sin [25,26], and it is part of the principle that the immune response will need some time to mature. The second serum collected following routine diagnostic procedures will be collected too early to see the new serotype-specific IgG becoming predominant. This is exactly what was observed in experiments with first and second sera from routine diagnostics at IPC. However, the effect of non-matching serotype-specificity was more conclusive in homologous neutralization assays than in assays using the strains from the reference panel. Moreover, we have tested sera collected several weeks after infection. In these experiments, we saw as expected a booster effect, but even after more than 14 days, the neutralization profile was identical to the neutralization profile from the early serum sample.

Strictly autologous serial challenges and neutralization studies are complicated to establish and ethically questionable. However, data from animal experiments showed clearly that serial challenges follow the rules of antigenic sin with antibody responses shifted to the new virus within 10 days [23]. Thus, in strictly autologous experiments, there is a clear picture that the immune system will develop antibodies shortly against the new strain after second or third virus challenge.

In contrast, a study on twenty monkeys vaccinated by the tetravalent vaccine CYD showed unique neutralization profiles (pre-challenge titers) after vaccination [27]. Each animal developed a specific pre-challenge antibody profile to the tetravalent CYD antigens. This antibody profile was boosted by the DENV challenge but was still of the same profile on day 28. For example, a vaccinated animal that showed the highest pre-challenge antibody titers against DENV-1 and -3 had the same profile after DENV-2 challenge. In these experiments, the vaccine-primed response was not always shifted to the challenge virus (13 of 20) [27].

Therefore, the effect of priming and boosting by heterologous or autologous DENV strains might cause one of the problems seen also in CYD-vaccinated people [28,29]. As an example, in 2019, the FDA approved the tetravalent CYD vaccine, but with a significant limitation. The vaccine was recommended only for those who were previously infected by DENV [30,31]. Under such naturally pre-primed conditions, the immune response developed against DENV can be boosted against all four serotypes by the vaccine. This is what will be expected and was also seen in our neutralization experiments. On the other hand, naïve individuals who are primed by the vaccine will be exposed to increased risk of more severe disease through ADE. This was an unexpected result first seen in young children but was later linked to the DENV unexposed status. The strains used for CYD were the classical laboratory-adapted reference strains and are each representative for one of the four DENV serotypes, but they are antigenetically very distant from actual DENV isolates. That is probably the reason why some of the DENV strains used for CYD did not provide optimal protection and safety to the vaccinees. The strains fulfill the classical serotype requirements such as genetic diversity but are antigenetically different to the on-site present DENV strains. Thus, a more effective vaccine should represent those DENVs actually circulating in endemic areas. Another aspect where false priming might play a role would be priming against DENV by asymptomatic infections [32,33] and traveling between areas where DENVs are antigenically different [34,35].

Virus-triggered antibodies are distinct from those created by vaccination, although distinguishing the two would require a new and complicated assay such as autologous neutralization assays, which might be useful to study DENV responses and neutralization in every detail. Such autologous experiments can be performed using patient isolates or pseudotyped virus. Pseudotyped viruses are ideal to study virus variation, escape from neutralization, or as part of gene delivery systems [36]. From RT-PCR-derived DNA or just sequence data, envelopes matching the patients virus and antibody response can be expressed and used to produce DENV- [37] or HIV-based pseudotypes [38]. Our data support once again the theory of original antigenic sin but also showed that an established neutralization profile to DENV-1-4 was not changed, or shifted toward the new infecting DENV strain. This effect was much better seen with homologous than heterologous DENV strains.

## Figures and Tables

**Table 1:** The three sets of dengue viruses used for the foci reduction neutralization test (FRNT^90^).

**REF**				
DENV-1	16007	Thailand	1964	Genbank AF180817
DENV-2	16681	Thailand	1984	Genbank U87411.1
DENV-3	H87	Philippines	1956	Genbank M93130
DENV-4	H241	Philippines	1956	Genbank AB609591
**IPC A**				
DENV-1	KH/BID-V2004/2006	Cambodia	2006	Genbank FJ639687
DENV-2	D2KH/06PHP	Cambodia	2006	IPC virus isolate
DENV-3	KH/BID-V2058/2005	Cambodia	2005	Genbank GQ868628
DENV-4	D4KH/98PHP	Cambodia	1998	IPC virus isolate
**IPC B**				
DENV-1	KH/BID-V2011/2007	Cambodia	2007	Genbank FJ639693
DENV-2	KH/BID-V2066/2007	Cambodia	2007	Genbank FJ639717
DENV-3	KH/BID-V2051/2007	Cambodia	2006	Genbank FJ639713
DENV-4	D4KH/07	Cambodia	2007	IPC virus isolate

REF, DENV reference strains; IPC A and IPC B, DENV isolated during the same epidemic season that the sera were collected; IPC, Institut Pasteur in Cambodia.

**Table 2:** Neutralization titers to DENV-1–4 reference strains using RT-PCR confirmed DENV sera.

		FRNT^90^, 1st Serum				FRNT^90^, 2nd Serum			
Serotype by		DENV				DENV			
RT-PCR		1	2	3	4	1	2	3	4
01	DENV-1	320	5120	640	2560	640	20,480	640	5120
02	DENV-1	20	320	40	160	1280	20,480	5120	5120
03	DENV-1	80	1280	80	640	320	5120	640	5120
04	DENV-1	40	320	40	320	1280	20,480	320	2560
05	DENV-1	<20	80	20	80	320	2560	160	2560
06	DENV-1	20	80	320	80	1280	2560	20,480	640
07	DENV-1	320	160	5120	320	320	320	5120	320
08	DENV-1	<20	<20	<20	<20	20	<20	<20	40
09	DENV-1	<20	<20	<20	<20	20	<20	<20	<20
10	DENV-1	20	20	20	20	80	640	80	640
11	DENV-2	5120	320	80	320	20,480	2560	1280	5120
12	DENV-2	160	40	20	40	20,480	2560	1280	2560
13	DENV-2	2560	640	80	320	10,240	5120	640	1280
14	DENV-2	1280	640	160	640	2560	1280	160	1280
15	DENV-2	40	2560	640	1280	80	2560	640	1280
16	DENV-2	160	1280	2560	1280	320	2560	5120	2560
17	DENV-2	160	1280	2560	1280	640	2560	20,480	2560
18	DENV-2	160	640	1280	1280	640	2560	10,240	2560
19	DENV-2	<20	160	<20	1280	640	160	40	320
20	DENV-2	40	1280	20	2560	80	2560	40	5120
21	DENV-3	5120	640	1280	320	5120	320	1280	320
22	DENV-3	5120	1280	320	640	5120	2560	320	2560
23	DENV-3	2560	640	320	320	10,240	2560	5120	5120
24	DENV-3	5120	2560	80	1280	5120	10,240	640	5120
25	DENV-3	160	2560	20	1280	1280	5120	40	2560
26	DENV-3	320	5120	160	2560	5120	20,480	1280	20,480
27	DENV-3	160	5120	160	2560	160	5120	320	5120
28	DENV-3	<20	320	<20	40	40	160	<20	40
29	DENV-3	640	5120	40	5120	640	5120	80	5120
30	DENV-3	320	10,240	320	5120	1280	5120	320	5120
31	DENV-4	1280	80	20	40	10,240	640	640	1280
32	DENV-4	320	40	<20	40	10,240	10,240	2560	1280
33	DENV-4	5120	160	160	80	20,480	1280	1280	640
34	DENV-4	640	20	20	40	10,240	2560	1280	1280
35	DENV-4	10,240	1280	640	1280	20,480	2560	1280	5120
36	DENV-4	40	1280	160	640	320	5120	2560	2560
37	DENV-4	640	20,480	320	5120	640	20,480	640	10,240
38	DENV-4	640	320	1280	80	20,480	2560	20,480	1280
39	DENV-4	160	160	5120	80	1280	640	20,480	640
40	DENV-4	640	1280	5120	2560	640	1280	20,480	2560

Marked in gray, the highest FRNT^90^ titer; Red frames, serotype matching with serotype-specific neutralization.

**Table 3:** Neutralizing specificity in sera from DENV-1–4 infected patients from Cambodia.

Serotype-Specific Neutralization	Number of Sera with Serotype by RT-PCR			
	DENV-1	DENV-2	DENV-3	DENV-4
DENV-1	4	20	7	11
DENV-2	10	3	9	7
DENV-3	7	7	-	5
DENV-4	3	4	-	-
DENV-1 + DENV-2	1	-	1	1
DENV-1 + DENV-3	2	-	-	1
DENV-2 + DENV-3	1	-	-	1
DENV-2 + DENV-4	6	-	4	-
DENV-3 + DENV-4	-	1	-	-
>2 serotypes	1	-	-	-
negative	2	-		-
Total number of sera	37	35	21	26

Red frames, number of matching results, -, negative.

**Table 4:** Neutralization titers in second sera from DENV-1-infected Cambodian patients.

Serum	FRNT^90^ *											
	REF				IPC A				IPC B			
	DENV				DENV				DENV			
	1	2	3	4	1	2	3	4	1	2	3	4
**24**	640	2560	<20	160	80	1280	640	<20	40	1280	640	20
**14**	80	2560	40	160	20	2560	320	<20	<20	2560	640	<20
**17**	80	5120	<20	320	<20	5120	1280	20	<20	5120	1280	<20
**25**	2560	10,240	80	320	80	5120	2560	<20	80	10,240	2560	20
**22**	160	5120	20	160	<20	10,240	320	20	<20	5120	640	40
**16**	80	640	2560	320	80	640	2560	<20	40	80	5120	<20
**23**	640	640	2560	640	40	640	5120	<20	20	80	5120	40
**30**	640	320	10,240	160	40	320	10,240	20	20	40	10,240	40
**15**	1280	320	10,240	160	40	320	20,480	20	40	320	20,480	160
**13**	<20	80	<20	40	<20	160	80	<20	<20	80	80	20
**03**	20	320	<20	160	<20	320	80	<20	<20	80	80	<20
**31**	320	1280	<20	320	<20	1280	160	20	<20	640	640	20
**33**	640	2560	<20	320	20	2560	320	<20	20	1280	1280	20
**01**	<20	80	<20	40	<20	80	40	<20	<20	<20	80	20
**29**	320	2560	<20	160	20	2560	640	<20	<20	320	1280	20
**21**	160	640	160	320	40	640	1280	<20	20	160	1280	20
**04**	320	640	<20	80	40	640	640	<20	40	160	640	<20
**08**	<20	160	<20	160	<20	320	160	<20	<20	80	160	<20
**20**	20	80	<20	1280	<20	80	320	20	<20	40	320	160
**19**	40	320	<20	1280	<20	320	320	40	<20	40	320	160
**09**	<20	<20	<20	20	<20	<20	<20	<20	<20	<20	<20	<20
**28**	160	160	<20	2560	<20	160	160	80	<20	40	320	640
**12**	20	80	<20	640	<20	80	160	20	<20	40	160	160
**05**	2560	640	40	80	640	640	640	<20	640	320	1280	20
**26**	640	640	<20	80	40	640	320	<20	40	320	640	20
**18**	640	640	640	320	20	640	5120	<20	20	80	1240	80
**07**	2560	640	20	80	160	320	1280	<20	320	320	1280	20
**11**	640	160	<20	80	20	160	320	<20	20	80	320	<20
**32**	2560	80	20	160	80	80	320	<20	40	20	1280	40
**06**	640	160	20	80	160	160	640	<20	160	40	1280	20
**10**	1280	80	20	80	20	40	1280	<20	20	80	1280	<20
**02**	640	320	40	640	40	320	1280	20	40	160	2560	40
**27**	640	320	320	640	40	160	2560	20	20	80	5120	20

Marked in gray, the highest FRNT^90^ titer; Red frames, serotype matching with serotype specific neutralization. *: it is same as Table 2.

**Table 5:** Serotype-specific neutralization in 33 DENV-1-positive Cambodian sera from Table 4.

Serotype-Specific Neutralization	Number of Sera		
	REF	IPC A	IPC B
DENV-1	6	-	-
DENV-2	13	13	5
DENV-3	4	15	21
DENV-4	5	-	1
DENV-1 + 2	1	-	-
DENV-1 + 4	2	-	-
DENV-2 + 3	-	3	4
DENV-2 + 4	1	-	-
DENV-3 + 4	-	-	1
>2 serotypes	1	1	-
negative	-	1	1
Total number of sera	33	33	33

Red frames, matching results for DENV-1 specificity; -, negative.

**Table 6:** Serotype specificity of antibodies in Colombian second sera P1−P7.

Colombian	Serotype	FRNT^90^	ED3 Dot Assay		ED3 ELISA		Days after
2nd Sera	RT-PCR	REF	REF	COL	REF	COL	Fever
P1	1	24	4	4	4	-	*16 d*
P2	1	2	2	2	-	-	*32 d*
P3	2	13	13	13	-	13	*24 d*
P4	2	123	123	123	1	123	*25 d*
P5	2	1	1	1	1	1	*21 d*
P6	4	12	123	12	-	123	*25 d*
P7	4	23	-	2	123	123	*37 d*

Numbers indicate DENV serotype; -, no serotype specificity was detected.

## Data Availability

Raw data to neutralization and antibody responses are given in our figures and tables.

## Appendix A

**Table A1:** Oligonucleotides for the cloning of COL MBP–ED3 fusion proteins.

**DENV-1**	
Oligo-1	5′-AAG GGG ATG TCA TAT GTG ATG TGC ACA GGC TCA TTT AAG CTA GAG AAG GAA GTG GCT GAG AC-3′
Oligo-2	5′-CGC GTC TGT TCC TTC GTA TTT GAC CTG CAC TAG AAC AGT TCC ATG CTG GGT CTC AGC CAC TTC CTT CTC T-3′
Oligo-3	5′-ACG AAG GAA CAG ACG CGC CAT GCA AGA TCC CCT TCT CGA CCC AAG ATG AGA AAG GAG TGA CCC AGA ATG-3‘
Oligo-4	5′-GAC TGA TTT TTC TTT GTC AGT AAC TAT GGG ATT GGC TGT TAT CAA TCT CCC ATT CTG GGT CAC TCC TTT C-3′
Oligo-5	5′-TTA CTG ACA AAG AAA AAT CAG TCA ACA TTG AGA CAG AAC CAC CTT TTG GTG AGA GCT ACA TCG TGG TAG GGG CAG-3′
Oligo-6	5′-GCT TCC TTT CTT GAA CCA GCT TAG TTT CAA GGC TTT TTC ACC TGC CCC TAC CAC GAT-3′
Oligo-7 BamHI-for	5′-CAC GGA TCC AAG GGG ATG TCA TAT GTG ATG TG-3′
Oligo-8 HindIII-rev	5′-CAC AAG CTT GCT TCC TTT CTT GAA CCA GCT-3′
**DENV-2**	
Oligo-1	5′-TCA TAC TCT ATG TGT ACA GGA AAG TTT AAA ATT GTG AGA GAA ATA GCA GAA ACA CAA CAT G-3′
Oligo-2	5′-CAT GGA GAA CCG TCC CCT TCA TAT TGT ATT CTG ATA ACT ATT GTT CCA TGT TGT GTT TCT GCT ATT TCT C-3′
Oligo-3	5′-GGG GAC GGT TCT CCA TGT AAG ATC CCT TTT GAA ATA ACA GAC TTG GAA AAA AGA CAC GTC TTA GGT CGC-3′
Oligo-4	5′-CTA TGT TGA CTG GGC TAT CTT TTT CTA TTA CGA TTG GGT TAA CTG TAA TCG GCG ACC TAA GAC GTG TCT TTT TT-3′
Oligo-5	5′-AAT AGA AAA AGA TAG CCC AGT CAA CAT AGA AGC AGA ACC TCC ATT CGG AGA CAG CTA CAT CAT CAT AGG AGT AGA G-3′
Oligo-6	5′-ACT TCC CTT CTT AAA CCA ATT GAG TTT CAA TTG TCC CGG CTC TAC TCC TAT GAT GAT GTA GCT GT-3′
Oligo-7 BamHI-for	5′-CAC GGA TCC TCA TAC TCT ATG TGT ACA GGA AAG TTT AAA-3′
Oligo-8 HindIII-rev	5′-GTG AAG CTT ACT TCC CCT CTT AAA CCA ATT GAG T-3′
**DENV-3**	
Oligo-1	5′-AAG GGG ATG AGC TAT GCA ATG TGC ACG AGT ACC TTT GTG TTG-3′
Oligo-2	5′-ATG AGT ATT GTC CCA TGT TGC GTT TCT GAG ACT TCT TTC TTC AAC ACA AAG GTA CTC GTG C-3′
Oligo-3	5′-CGC AAC ATG GGA CAA TAC TCA TCA AGG TCG AGT ACA AAG GGG AAG ATG TAC CTT GCA AG-3′
Oligo-4	5′-TGT GAG CTT TCC CTT GTC CAT CCT CTG TGG AGA AAG GAA TCT TGC AAG GTA CAT CTT CCC C-3‘
Oligo-5	5′-TGG ACA AGG GAA AGC TCA CAA TGG CAG ACT GAT TAC AGC CAA CCC AGT GGT GAC TAA GAG-3′
Oligo-6	5′-CCA AAA GGA GGT TCA GCC TCA ATA TTG ACA GGC TCC TCC CTC TTA GTC ACC ACT GGG TTG-3′
Oligo-7	5′-TGA GGC TGA ACC TCC TTT TGG GGA AAG TAA TAT AGT AAT TGG AAT TGG AGA CAA CGC CTT-3′
Oligo-8	5′-GCT TCC CTT CTT GTA CCA GTT GAT TTT CAA GGC GTT GTC TCC AAT TCC-3′
Oligo-9 BamHI-for	5′-CACGGATCCA AGGGGATGAG CTATGCAATG-3′
Oligo-10 HindIII-rev	5′-GTGAAGCTTG CTTCCCTTCT TGTACCAGTT G-3′
**DENV-4**	
Oligo-1	5′-AAA GGT ATG TCT TAC ACG ATG TGT TCG GGC AAG TTT AGT ATT GAT AAA GAA ATG GCG GAA ACG-3′
Oligo-2	5′-GGG GGC TCC CGC CCC TTC GTA TTT GAC TTT CAC CAC TGT CGT TCC GTG CTG CGT TTC CGC CAT TTC TTT ATC-3′
Oligo-3	5′-CGA AGG GGC GGG AGC CCC CTG TAA AGT CCC CAT TGA AAT TCG GGA CGT GAA CAA GGA GAA AGT CGT AGG-3′
Oligo-4	5′-CTA TAT TCG TGA CAC TGT TCG TAT TTT CTG CCA GAG GTG TTG CAG AGA TCA CAC GTC CTA CGA CTT TCT CCT TGT TC-3′
Oligo-5	5′-CGA ACA GTG TCA CGA ATA TAG AAC TTG AAC CGC CCT TTG GCG ACA GCT ATA TAA TGA TAG GGG TGG GC-3′
Oligo-6	5′-CGA TCC TTT TCT GAA CCA ATG AAG GGT TAA TGC AGA ATT G CC CAC CCC TAT CAT TAT ATA GC-3′
Oligo-7 BamHI-for	5′-CAC GGA TCC AAA GGT ATG TCT T-3′
Oligo-8 HindIII-rev	5′-CAC AAG CTT CGA TCC TTT TCT G-3′
